# Structural brain MRI studies in autism spectrum disorder

**DOI:** 10.1192/j.eurpsy.2023.1934

**Published:** 2023-07-19

**Authors:** J. M. Petrović, I. Binic, A. Stojanovic, M. Zdravkovic, F. Petrovic

**Affiliations:** 1 Psychiatry, Special Hospital for Psychiatic Diseases “Gornja Toponica”, Nis, Serbia; 2 Psychiatry, Psychiatry Clinic University Clinical Center Nis; 3 Child and adolescent psychiatry, Center for Mental Health Protection University Clinical Center Nis; 4 Forensic medicine, Depertment of Forensic Medicine Medical Faculty University of Nis; 5 Radiology, Radiology Center, University Clinical Center Nis, Nis, Serbia

## Abstract

**Introduction:**

Autism spectrum disorder (ASD) refers to a group of conditions characterized by quantitative differences in the morphology of the cortex and subcortex. Analyzing brain morphology qualitatively provides complementary information about possible underlying neurobiology. Studies of neuroradiological findings in ASD have produced mixed results in a large and independent sample.

**Objectives:**

A small cerebellum associated with pons hypoplasia, or a posterior fossa cyst, may indicate causal developmental mechanisms. Therefore, neuroradiological findings could help elucidate the neurodevelopmental processes associated with ASD.

MRI “minor abnormalities” also included dilatation of the Virchow-Robin gaps, an enlarged cisterna magna, pineal gland cysts, and arachnid or choroidal cysts not included in specified categories.

**Methods:**

There were anomalies in the corpus callosum (hypoplasia), cerebellum, brain stem, abnormal white matter signal intensity, macrocephaly, ventriculomegaly, abnormal myelination patterns, ventricular system size, Arnold Chiari I malformation, cortical dysplasia and atrophy, hippocampal malformations, and pituitary glands. These anomalies were referred to as “major abnormal findings”.

**Results:**

The most common minor abnormality is the mega cisterna magna. Some authors propose a minor abnormality such as this as a marker for brain dysgenesis. According to Zimmer and colleagues, enlargements of the cisterna magna are generally accompanied by cerebellar hypoplasia and ventriculomegaly, as well as lower performance on speech tasks (verbal and semantic fluency) common among individuals with autism spectrum disorders. The relationship between the presence of mega cisterna magna and language difficulties could be studied further in a subsequent study. Abnormal dilation of the cisterna magna is thought to be related to alterations in the cerebellar volumes.

**Conclusions:**

Clinical MRI assessments may be helpful in the context of diagnoses and are potentially valuable for further studies of the pathogenesis of autism. The potential utility of routine brain MRI is in discovering early morphologic biomarkers for ASD.

**Disclosure of Interest:**

None Declared

